# Enhanced HBsAg Synthesis Correlates with Increased Severity of Fibrosis in Chronic Hepatitis B Patients

**DOI:** 10.1371/journal.pone.0087344

**Published:** 2014-01-31

**Authors:** Mei-Zhu Hong, Wen-Qi Huang, Feng Min, Jin-Chao Xu, Zhen Lin, Kuang-Nan Fang, Jin-Shui Pan

**Affiliations:** 1 Department of Infectious Diseases, Chenggong Hospital affiliated to Xiamen University (The 174th Hospital of PLA), Xiamen, China; 2 Department of Pathology, Chenggong Hospital affiliated with Xiamen University (The 174th Hospital of PLA), Xiamen, China; 3 Department of Statistics, School of Economics, Xiamen University, Xiamen, China; 4 Department of Gastroenterology, Zhongshan Hospital affiliated with Xiamen University, Xiamen, China; Harvard Medical School, United States of America

## Abstract

**Background and Aims:**

Little is known about whether low serum HBsAg levels result from impaired HBsAg synthesis or a reduced number of hepatocytes caused by advanced liver fibrosis. Therefore, we investigated the capacity for HBsAg synthesis in a cross-sectional cohort of treatment-naïve chronic hepatitis B patients.

**Methods:**

Chronic hepatitis B patients (n = 362) were enrolled; liver biopsies were performed and liver histology was scored, and serum HBsAg and HBV DNA levels were investigated. In the enrolled patients, 183 out of 362 have quantitative serum HBsAg levels. Tissue HBsAg was determined by immunohistochemistry.

**Results:**

A positive correlation between serum HBsAg and HBV DNA levels was revealed in HBeAg(+) patients (r = 0.2613, *p* = 0.0050). In HBeAg(+) patients, serum HBsAg and severity of fibrosis were inversely correlated (*p* = 0.0094), whereas tissue HBsAg levels correlated positively with the stage of fibrosis (*p* = 0.0280). After applying the mean aminopyrine breath test as a correction factor, adjusted serum HBsAg showed a strong positive correlation with fibrosis severity in HBeAg(+) patients (r = 0.5655, *p*<0.0001). The adjusted serum HBsAg values predicted ‘moderate to severe’ fibrosis with nearly perfect performance in both HBeAg(+) patients (area under the curve: 0.994, 95% CI: 0.983–1.000) and HBeAg(−) patients (area under the curve: 1.000, 95% CI: 1.000–1.000).

**Conclusions:**

Although serum HBsAg levels were negatively correlated with fibrosis severity in HBeAg(+) patients, aminopyrine breath test-adjusted serum HBsAg and tissue HBsAg, two indices that are unaffected by the number of residual hepatocytes, were positively correlated with fibrosis severity. Furthermore, adjusted serum HBsAg has an accurate prediction capability.

## Introduction

Chronic hepatitis B (CHB) remains one of the most common infectious diseases worldwide, and in Asia and Africa in particular. Interferon α (or pegylated-interferon) and nucleosides (or nucleotide analogues) are prescribed widely for the treatment of CHB. Following effective therapy, a marked decline in hepatitis B virus (HBV) DNA load and/or quantified serum hepatitis B surface antigen (HBsAg) can be observed. There is a positive correlation between HBV DNA level and liver cirrhosis and hepatocellular carcinoma (HCC) [1], [2]. In the past several years, the role of quantified serum HBsAg in the surveillance of treatment efficacy has attracted interest. HBsAg seroclearance confers a favorable prognosis in cirrhosis and HCC [3]–[6]. The risk of hepatocellular tumorigenesis is lower in the patients with clearance of serum HBsAg after interferon α-based antiviral therapy [7]. High levels of HBsAg increase the risk of HCC and vice versa [8]. Moreover, a clinical association between serum HBsAg quantification and liver histology has been reported [9], [10].

Recently, two studies have reported on the negative correlation between serum HBsAg level and the severity of liver fibrosis [11], [12]. However, the number of viable hepatocytes and the capacity for synthesis of HBsAg critically affect serum HBsAg levels. Therefore, it is not clear whether the diminished serum HBsAg levels in patients with advanced fibrosis are due to a decrease in the number of residual hepatocytes or to impaired secretion of HBsAg, and the results of these two studies may have been misinterpreted in terms of the beneficial effect of HBsAg in the control of liver fibrosis. Furthermore, these studies are based on cross-sectional observations, yielding insufficient information on the role of serum HBsAg in liver fibrosis. Thus, clinical investigation is necessary to explore the cause of diminished serum HBsAg in the patients with advanced liver fibrosis.

HBsAg is secreted into the circulation as tubular forms or spherical particles by HBV-infected hepatocytes. Patients with CHB usually express different levels of HBsAg when assayed by immunohistochemistry. In patients with advanced fibrosis, residual hepatocytes diminish dramatically, which has a profound effect on serum HBsAg quantification, but not on intrahepatic HBsAg (tissue HBsAg) quantification. Tissue HBsAg levels are determined by the establishing the percentage of HBsAg-positive hepatocytes and the grade of staining reflected by immunohistochemistry. Therefore, the number of hepatocytes has limited effects on tissue HBsAg levels.

Research by Herold *et al.* [13] reported on the use of the aminopyrine breath test (ABT), which measures the metabolic capacity of hepatocytes, in a series of patients with different stages of fibrosis and grades of inflammatory activity. Increasing fibrosis or inflammatory activity was associated with decreasing ABT values. Thus, the ABT value may reflect the number of hepatocytes to some degree. In order to rule out the effect of the number of hepatocytes on serum HBsAg levels, the present study aims at investigating the correlation between ABT adjusted serum HBsAg and fibrosis in a large cohort of treatment-naïve CHB patients. The potential relationship between intrahepatic HBsAg and the severity of liver histology will be also discussed.

## Materials and Methods

### Patients

Treatment-naïve CHB patients reporting to the 174^th^ Hospital of the PLA in Fujian, China, between 2010 and 2013 were enrolled. Patients were investigated retrospectively and informed consent was obtained from all the patients. The present study was approved by the Institutional Ethics Board of Chenggong Hospital (the 174^th^ Hospital of the PLA) and Zhongshan Hospital, Xiamen University. All patients provided written consent prior to liver biopsy and study entry. Entire study was conducted according to the principles of the Declaration of Helsinki. Inclusion criteria for patients were: HBsAg-positive, known HBeAg status, and scheduled liver biopsy. Patients were excluded in the following situations: Hepatitis C virus, Hepatitis D virus, or Human Immunodeficiency Virus co-infection, significant steatosis, alcoholic fatty liver, and decompensated cirrhosis. A total of 362 treatment-naïve CHB patients were included in the present study.

Liver biopsies were performed on the same day that serum samples were collected, or in less than 2 days thereafter.

### Diagnostic Tests

HBsAg levels were assayed using the HBsAg assay kit (Wantai Biological Co., Beijing, China). HBV DNA levels were determined by quantitative fluorescence PCR on an ABI 7000 (Applied Biosystems, Carlsbad, USA), with a lower limit of detection of 500 IU/ml. HBsAg and HBV DNA were expressed as log IU/ml. Serum ALT and AST levels were expressed as IU/ml. Liver histology was assessed using the Scheuer scoring system [14]. Liver histology was evaluated by two independent pathologists who were blind to the study design. If the two pathologists could not agree on the pathological diagnosis, histological scores were made and confirmed by a panel of pathologists. Intrahepatic HBsAg was determined by immunohistochemistry. The primary antibody for HBsAg was purchased from Maixin Company (catalog no. MAB-0587; Fuzhou, China). Grade of tissue HBsAg was defined according to the percentile of anti-HBsAg staining hepatocytes, as per the following metrics: 0+, 0∼1.9%; 1+, 2.0∼24.9%; 2+, 25∼49.9%; 3+, 50∼74.9%; 4+, 75∼100%.

### Data Analysis

Quantitative testing of liver functions in a large sample of fibrosis patients related to chronic hepatitis have been reported by Herold *et al.* [13]. Since the investigated objects in our study were patients with CHB, it seems reasonable that mean ABT values in our study is comparable to that of Herold *et al*. Mean values of ABT in patients with different stages of fibrosis and grades of inflammatory activity have been shown by Herold *et al*. Gm is the grade of inflammatory activity and Sn is the stage of fibrosis of a group of CHB patients. According to the Scheuer scoring system, inflammatory grade and fibrosis stage were scored as 0, 1, 2, 3, or 4. Thus, Gm represented anyone of G0, G1, G2, G3 or G4 while Sn represented anyone of S0, S1, S2, S3 or S4. If the liver histology of a group of patients was GmSn, the mean value of ABT of this group, ABT(GmSn), was calculated from the data provided by Herold *et al.* [13] by using the following expression: ABT(GmSn) = ABT(Gm) × ABT(Sn).

Statistical analyses were performed using R platform (version 3.0.1, http://www.R-project.org) or GraphPad Prism 6.01. Quantitative data conformed to Gaussian distribution were tested using ANOVA. Qualitative data or quantitative data that did not pass a Gaussian distribution test were analyzed using a nonparametric test, such as the Kruskal-Wallis test. The correlation of quantitative data was determined by the Pearson correlation test. Correlation of ordinal data was tested using the Spearman rank correlation test. All tests were two-sided and *p*<0.05 was regarded as statistically significant. HBV DNA and HBsAg levels were logarithmically transformed. For patients with HBV DNA levels lower than 500 IU/ml, a value of 250 IU/ml was assigned.

## Results

### Patients’ Characteristics

**Figure 1:**
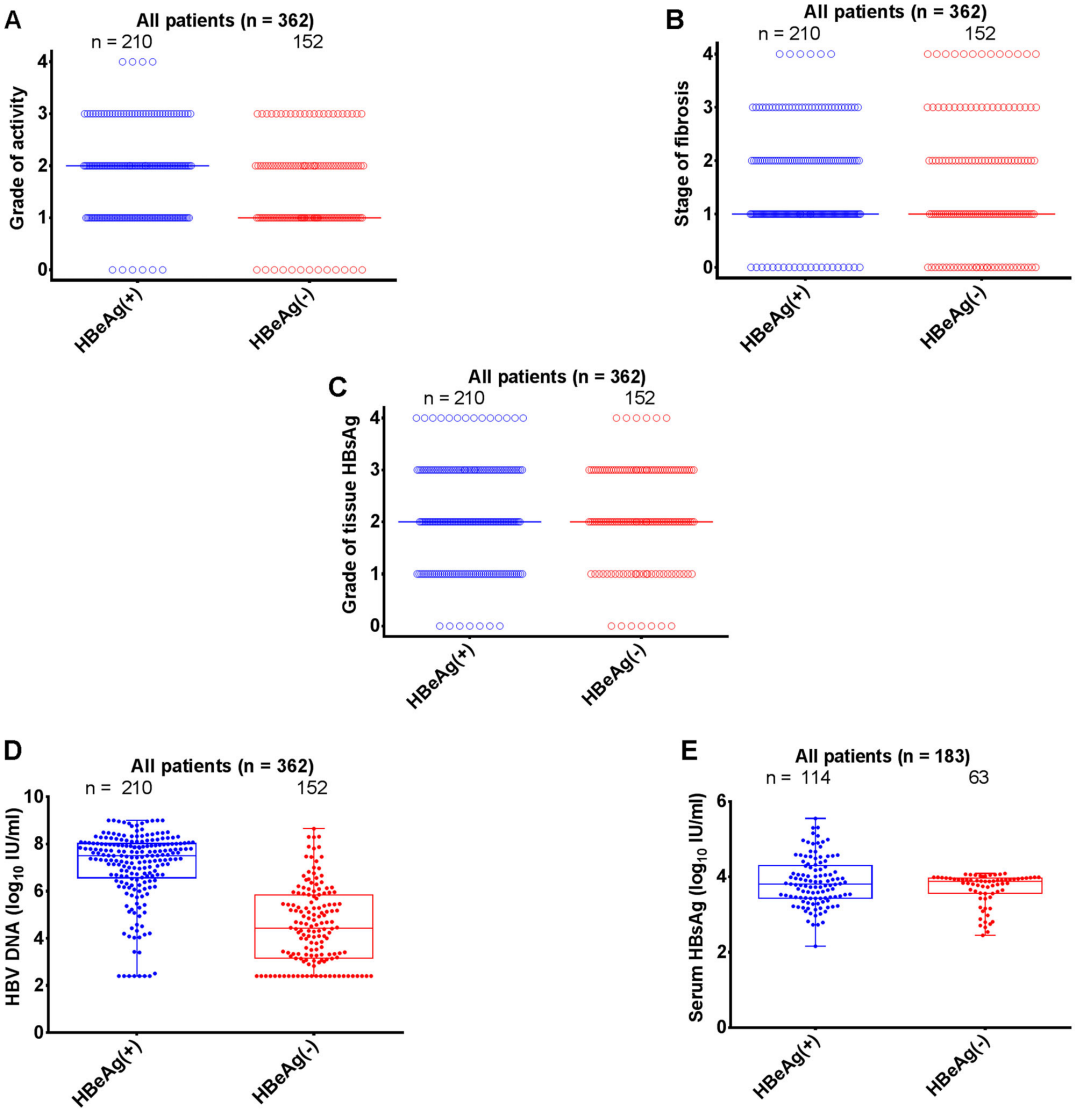
Patients’ characteristics stratified according to HBeAg status. (A) grade of inflammatory activity; (B) stage of fibrosis; (C) immunohistochemistry grade of tissue HBsAg; (D) HBV DNA (log10 IU/ml); (E) serum HBsAg (log10 IU/ml).

Among the 362 enrolled patients, 210 (58.0%) were HBeAg(+) and 152 (42.0%) were HBeAg(−) (Table 1). In both HBeAg(+) and HBeAg(−) groups, there were more men (n = 285; 78.7%) than women. Patients who were HBeAg(+) tended to younger than HBeAg(−) patients (*p*<0.0001). The mean alanine aminotransferase (ALT) levels and mean aspartate aminotransferase (AST) levels were significantly higher in HBeAg(+) patients than in HBeAg(−) patients (*p*<0.0001). Proportions of patients with normal ALT or AST in the HBeAg(−) group were significantly greater than in the HBeAg(+) group (*p*<0.0001). The grade of inflammation activity observed in HBeAg(+) patients was significantly higher compared with that seen in HBeAg(−) patients (median:2.0 vs. 1.0, Mann Whitney test, *p* = 0.0018; Fig. 1A). However, there was no statistically significant difference regarding the stage of fibrosis between the two groups (median: 1.0 vs. 1.0, Mann Whitney test, *p* = 0.6253; Fig. 1B). The expression of tissue HBsAg was similar in both groups (median: 2.0 vs. 2.0, Mann Whitney test, *p* = 0.5703; Fig. 1C).

**Table 1:** Patient characteristics.

Patient group	All(N = 362)	HBeAg(+) (n = 210)	HBeAg(−) (n = 152)	*p* value*
Age, yr (Mean ± SD)	35.5±9.9	32.7±8.7	40.2±9.6	<0.0001
Male, n (%)	285 (78.7)	160 (76.2)	125 (82.2)	0.1935
ALT, IU/L (Mean ± SD)	144.7±207.0	176.6±236.9	100.2±145.2	<0.0001
Normal ALT, n (%)	62 (17.1)	19 (9.0)	43 (28.3)	<0.0001
AST, IU/L (Mean ± SD)	77.0±111.9	92.6±133.2	55.5±67.6	<0.0001
Normal AST, n (%)	163 (45.0)	73 (34.8)	90 (59.2)	<0.0001
**Liver histology** †				
Activity, n (%)				
G0	19 (5.2)	6 (2.9)	13 (8.6)	0.0290
G1	145 (40.1)	75 (35.7)	70 (46.0)	0.0511
G2	132 (36.5)	86 (40.9)	46 (30.3)	0.0463
G3	62 (17.1)	39 (18.6)	23 (15.1)	0.4800
G4	4 (1.1)	4 (1.9)	0 (0)	0.1423
**Fibrosis, n (%)**				
S0	53 (14.6)	21 (10.0)	32 (21.1)	0.0041
S1	152 (42.0)	99 (47.1)	53 (34.9)	0.0234
S2	84 (23.2)	51 (24.3)	33 (21.7)	0.6148
S3	54 (15.0)	33 (15.7)	21 (13.8)	0.0083
S4	19 (5.2)	6 (2.9)	13 (8.5)	0.0290
**Tissue HBsAg, n (%)**				
0	14 (3.9)	7 (3.3)	7 (4.6)	0.5875
1	83 (22.9)	55 (26.2)	28 (18.4)	0.0905
2	134 (37.0)	73 (34.8)	61 (40.1)	0.3217
3	111 (30.7)	61 (29.0)	50 (32.9)	0.4886
4	20 (5.5)	14 (6.7)	6 (4.0)	0.3526
HBV DNA, log_10_ IU/ml (Mean ± SD)	6.01±1.98	7.07±1.49	4.55±1.63	<0.0001

* HBeAg(+) vs. HBeAg(−);

† According to the Scheuer scoring system.

The study cohort was analyzed both as a whole and stratified according to HBeAg status.

All patients underwent an HBV DNA assay. However, due to cost, quantitative HBsAg measurement was performed in only 183 of the 362 enrolled patients. Detailed informations of the 183 patients were listed in Table 2. HBeAg(+) patients exhibited significantly higher serum levels of HBV DNA compared with HBeAg(−) patients (*p*<0.0001; Fig. 1D). HBeAg(+) patients tended to have higher serum HBsAg levels than did HBeAg(−) patients, although there was no statistically significant difference between the two groups (mean ± SD: 3.87±0.64 vs. 3.68±0.44, *p* = 0.2600; Fig. 1E).

**Table 2:** Characteristics of the 183 patients with quantitative serum HBsAg levels.

Patient group	All (N = 183)	HBeAg(+) (n = 113)	HBeAg(−) (n = 69)	*p* value*
Age, yr (Mean ± SD)	34.7±10.2	31.0±8.1	40.9±10.3	<0.0001
Male, n (%)	147 (80.3)	86 (75.4)	61 (88.4)	0.0358
ALT, IU/L (Mean ± SD)	143.2±199.6	168.3±220.0	101.1±152.0	<0.0001
Normal ALT, n (%)	30 (16.4)	10 (8.8)	20 (29.0)	0.0007
AST, IU/L (Mean ± SD)	72.8±92.8	83.8±104.9	54.3±64.4	<0.0001
Normal AST, n (%)	87 (47.5)	46 (40.7)	41 (59.4)	0.0152
**Liver histology** †				
Activity, n (%)				
G0	10 (5.5)	4 (3.5)	6 (8.7)	0.1824
G1	75 (41.0)	45 (39.5)	30 (43.5)	0.6444
G2	67 (36.5)	47 (41.2)	20 (29.0)	0.1130
G3	27 (14.8)	14 (12.3)	13 (18.8)	0.2837
G4	4 (2.2)	4 (3.5)	0 (0)	0.2990
Fibrosis, n (%)				
S0	25 (13.6)	14 (12.3)	11 (15.9)	0.5127
S1	81 (44.3)	55 (48.2)	26 (37.7)	0.1681
S2	38 (20.8)	26 (22.8)	12 (17.4)	0.4531
S3	26 (14.2)	14 (12.3)	12 (17.4)	0.3864
S4	13 (7.1)	5 (4.4)	8 (11.6)	0.0807
**Tissue HBsAg, n (%)**				
0	6 (3.3)	3 (3.3)	3 (4.6)	0.6748
1	40 (21.9)	27 (26.2)	13 (18.4)	0.4654
2	64 (35.0)	36 (34.8)	28 (40.1)	0.2641
3	59 (32.2)	37 (29.0)	22 (32.9)	1.0000
4	14 (7.6)	11 (6.7)	3 (4.0)	0.2553
HBsAg, log_10_ IU/ml (n, Mean ± SD)	183, 3.80±0.58	114, 3.87±0.64	69, 3.68±0.44	0.2600
HBV DNA, log_10_ IU/ml (Mean ± SD)	6.05±2.08	7.17±1.45	4.20±1.58	<0.0001

† According to the Scheuer scoring system.

The study cohort was analyzed both as a whole and stratified according to HBeAg status.

### Positive Correlation between Serum HBsAg and HBV DNA Levels

**Figure 2:**
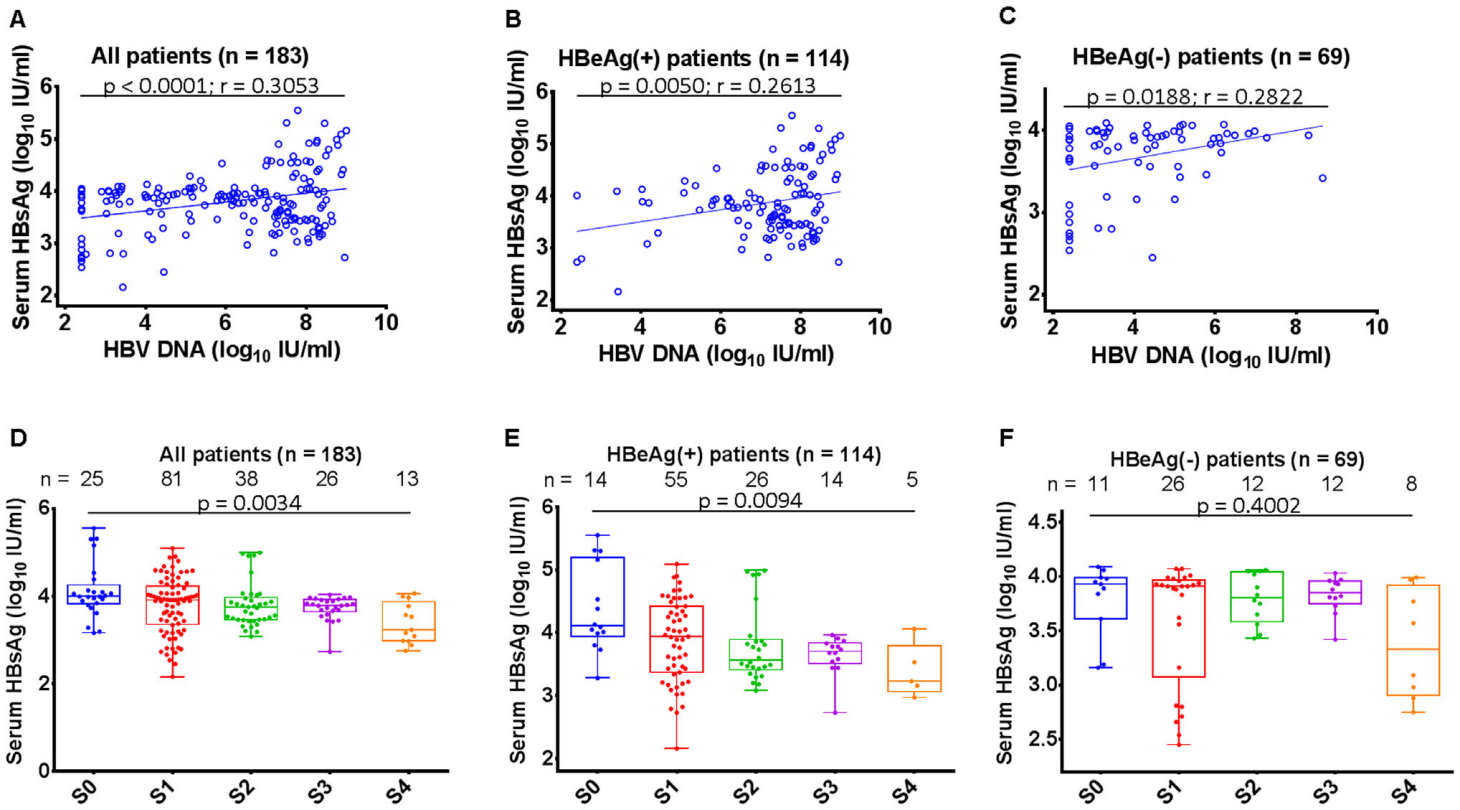
Correlation between serum HBsAg and HBV DNA and stage of fibrosis in treatment-naïve CHB patients. (A–C) correlation between serum HBsAg (log10 IU/ml) and HBV DNA (log10 IU/ml) in 183 patients with quantified serum HBsAg values, HBeAg(+) patients, and HBeAg(−) patients, respectively. (D–F) correlation between serum HBsAg (log10 IU/ml) and stage of fibrosis in 183 patients with quantified serum HBsAg values, HBeAg(+) patients, and HBeAg(−) patients, respectively.

In the 183 patients in whom serum HBsAg levels were quantified, there was a positive correlation between serum HBsAg and HBV DNA levels (r = 0.3053, *p*<0.0001; Fig. 2A). After stratifying the patients according to HBeAg status, a correlation was found between HBsAg and HBV DNA levels in HBeAg(+) patients (r = 0.2613, *p* = 0.0050; Fig. 2B) as well as in HBeAg(−) patients (r = 0.2822, *p* = 0.0188; Fig. 2C).

### Negative Correlation between Serum HBsAg Levels and Stage of Fibrosis

Severity of liver fibrosis was scored using the Scheuer system [14]. In the 183 patients with quantitative HBsAg data, increasing severity of fibrosis was associated with a decreasing serum HBsAg (*p* = 0.0034; Fig. 2D) and there was a negative correlation (r = −0.2711; *p* = 0.0002). In HBeAg(+) patients, this negative relationship was also significant (*p* = 0.0094; Fig. 2E) and the correlation was (r = −0.3212; *p* = 0.0005); however, no confirmed relationship could be seen in HBeAg(−) patients (*p* = 0.4002; Fig. 2F).

### Positive Correlation between ABT-adjusted Serum HBsAg Levels and Stage of Fibrosis

Table 3 was deduced from the data reported in Herold *et al.* [13]. ABT-adjusted serum HBsAg was calculated by dividing serum HBsAg (log_10_ IU/ml) by ABT(GmSn).

**Table 3:** Mean value of aminopyrine breath test (ABT) in different stage of fibrosis and grade of activity.

	S0*	S1	S2	S3	S4
G0	0.55	0.40	0.42	0.24	0.21
G1	0.52	0.38	0.40	0.22	0.19
G2	0.47	0.34	0.36	0.20	0.17
G3	0.48	0.35	0.37	0.21	0.18
G4	0.43	0.31	0.33	0.19	0.16

* Herold *et al*. [13] did not provided the mean value of ABT in S0. The mean value of ABT in the control group was used as the mean value of ABT in S0.

**Figure 3:**
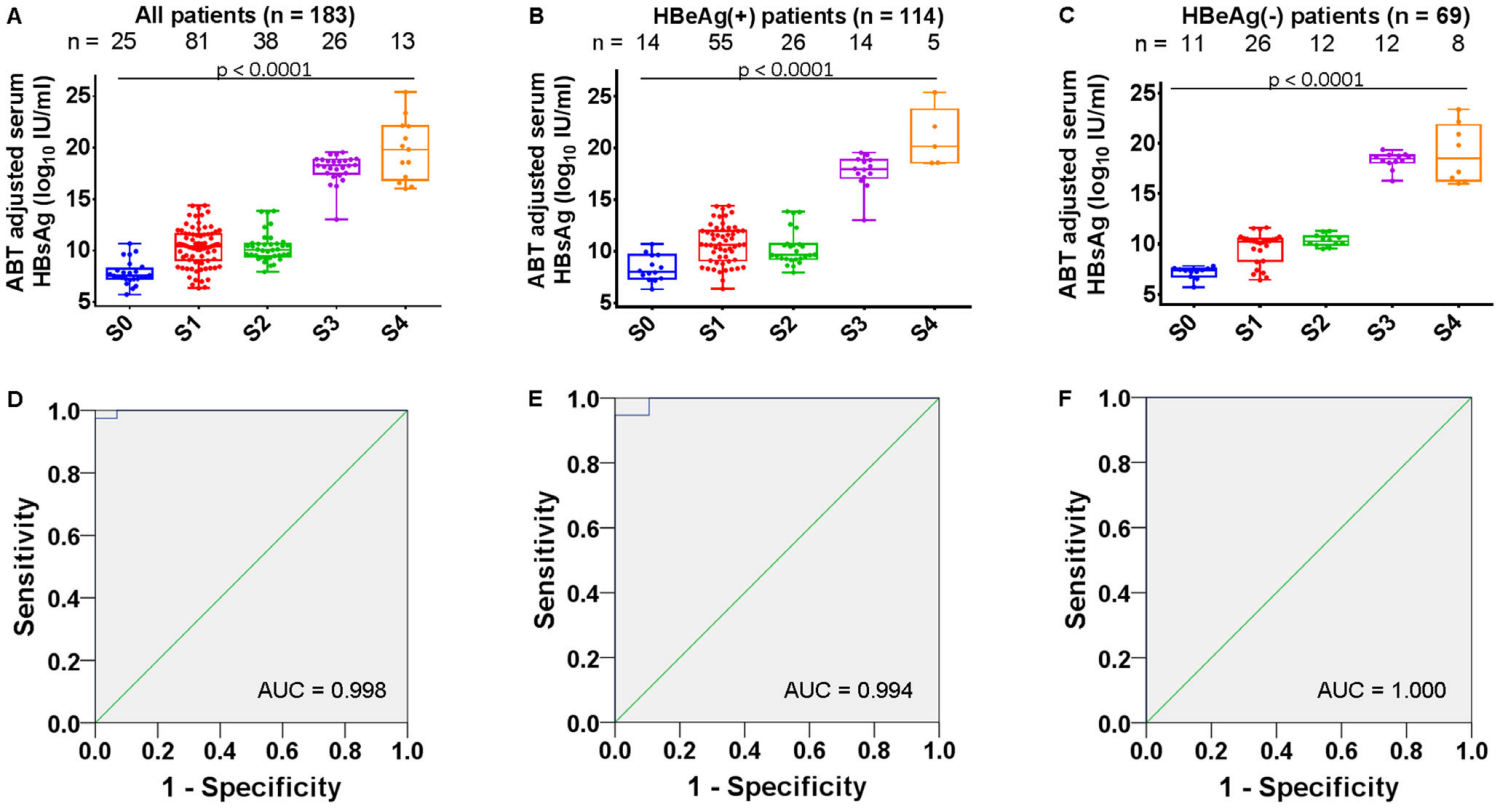
Correlation between aminopyrine breath test (ABT)-adjusted serum HBsAg and fibrosis severity. (A–C) correlation between ABT-adjusted serum HBsAg (log10 IU/ml) and fibrosis severity in 183 patients with quantified serum HBsAg values, HBeAg(+) patients and HBeAg(−) patients, respectively. Stage of fibrosis was determined according to the Scheuer scoring system. (D–F) diagnostic performance (AUC) of aminopyrine breath test -adjusted serum HBsAg in recognizing moderate to severe fibrosis (S3 and S4) in all of 183 patients, HBeAg(+) patients, and HBeAg(−) patients, respectively.

In all 183 patients, or when stratifying this cohort into HBeAg(+) and HBeAg(−) patients, increasing ABT-adjusted serum HBsAg values were associated with increasing severity of fibrosis (Fig. 3A, 3B, 3C, respectively). Specifically, in the 183 patients, a positive correlation was seen between severity of fibrosis and ABT-adjusted serum HBsAg values (r = 0.6929, *p*<0.0001); a similar phenomenon was observed in HBeAg(+) patients (r = 0.5655, *p*<0.0001), while in HBeAg(−) patients the correlation was even stronger (r = 0.8289, *p*<0.0001).

### Role of ABT-adjusted Serum HBsAg Levels in Differentiating CHB Patients with no to Mild Fibrosis from those with Moderate to Severe Fibrosis

Stages S0–S1 fibrosis were defined as ‘no to mild’, while stages S3–S4 were defined as ‘moderate to severe’ fibrosis. In order to evaluate the diagnostic performance of ABT-adjusted serum HBsAg levels in differentiating patients with S0–S1 fibrosis from patients with S3–S4 fibrosis, ROC curves were generated. In the 183 patients with quantified serum HBsAg levels, the ABT-adjusted serum HBsAg values had a nearly perfect area under the curve (AUC) for differentiating stages S0–S2 from stages S3–S4 (AUC: 0.998, 95% confidence interval [CI]: 0.994–1.000; Fig. 3D). For HBeAg(+) patients, the AUC was 0.994 (95% CI: 0.983–1.000; Fig. 3E), and in HBeAg(−) patients AUC was 1.000 (95% CI: 1.000–1.000; Fig. 3F). In the 183 patients, a cut-off value for ABT-adjusted serum HBsAg (log_10_ IU/ml) of 15.0 had a sensitivity of 97.44%, a specificity of 100.00%, and a positive predictive value of 100.00%, with a negative predictive value of 99.31% for recognizing stages S3–S4 of fibrosis.

### Positive Correlation between Tissue HBsAg Levels and Severity of Fibrosis

**Figure 4:**
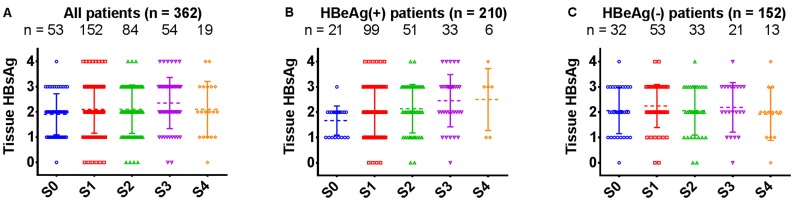
Expression of tissue HBsAg in patients with different stages of fibrosis. (A–C) grade of tissue HBsAg in CHB patients stratified according to the stage of fibrosis in all enrolled patients, HBeAg(+) patients, and HBeAg(−) patients, respectively. Stage of fibrosis was determined according to the Scheuer scoring system.

When stratifying the enrolled 362 patients according to the severity of fibrosis, there was a trend toward increasing tissue HBsAg levels with increasing fibrosis severity in HBeAg(+) patients (*p* = 0.0040), as is shown in Fig. 4B. However, no confirmed relationship between tissue HBsAg and severity of fibrosis was observed in the total group of 362 patients (*p* = 0.1618; Fig. 4A), or in HBeAg(−) patients (*p* = 0.7067; Fig. 4C). A positive correlation was revealed between tissue HBsAg levels and fibrosis in the group of 362 patients (Spearman rank correlation, r = 0.1092, *p* = 0.0378; Fig. S1A). In HBeAg(+) group, the positive correlation was even stronger (r = 0.2168, *p* = 0.0016; Fig. S1B), but there was no statistically significant correlation in the HBeAg(−) group (*p* = 0.7786; Fig. S1C).

## Discussion

In brief, we preformed this cross-sectional study in a group of 362 unselected CHB patients and attempted to elucidate the relationship between serum HBsAg or tissue HBsAg and severity of fibrosis. In China, due to medical insurance cover, some of the patients accepted qualitative measurement of HBsAg. Thus, quantitative HBsAg levels could be determined in only 183 patients. An investigation performed by Zeng *et al.* [15] indicated that genotype B and genotype C of HBV accounted for up to 94% of HBV infections in Chinese patients. We could not recruit sufficient patients infected with genotypes other than B and C for statistical analysis, and therefore, HBV genotyping was not performed.

We observed that HBeAg(−) patients tended to have a higher percentage of normal ALT or AST levels, lower HBV DNA load, with mild necroinflammation, as compared to HBeAg(+) patients. Loss of HBeAg may indicate that the hosts had attained a certain level of immune-control over HBV and these hosts may become ‘inactive carriers’ [16], [17], which may partly explain the aforementioned phenomena. However, the severity of fibrosis in HBeAg(−) patients was similar to that in HBeAg(+) patients. Since HBeAg(−) patients tended to be older than HBeAg(+) patients, the former may have undergone repeated bouts of inflammation than the latter, although the necroinflammation was milder in HBeAg(−) patients.

In the past several years, increasing numbers of studies have been performed to establish the role of serum HBsAg quantification in the prediction of therapy response [18], [19], intervention monitoring [20], [21], and disease progression [22]. Quite recently, the relationship between serum HBsAg and liver histology has been discussed [9]–[12]. In the present study, a positive correlation between serum HBsAg and HBV DNA load was revealed in the treatment-naïve HBeAg(+) CHB patients. In HBeAg(−) CHB patients, there tended to be a similar but weaker relationship, which was in accordance with previous studies [23]–[25].

In treatment-naïve CHB patients, pre-core promoter mutations may lead to impaired secretion of HBeAg and HBV virions [26], which are the main source of serum HBV DNA, while pre-S region mutations would reduce serum HBsAg output [27], [28]. Since these mutations do not always occur simultaneously, unbalanced production of HBsAg and HBV DNA may be observed.

Similar to previous reports [9]–[12], we also observed a strong inverse correlation between serum HBsAg and severity of fibrosis in HBeAg(+) CHB patients, whereas this correlation was not evident in HBeAg(−) patients (Fig. 2E, F). Interestingly, after adjusted by mean ABT (listed in Table 3), the ABT-adjusted serum HBsAg values highly positively correlated with the severity of fibrosis in both HBeAg(+) and HBeAg(−) CHB patients (Fig. 3B, C). ABT reflects the metabolic capacity of hepatocytes and essentially mirrors the quantity of viable hepatocytes. Therefore, ABT-adjusted serum HBsAg values would be unaffected by variation in the number of hepatocytes and could be regarded as a way to map the amount of HBeAg secreted by a ‘single hepatocyte’.

As for the prediction of severity of fibrosis, ABT-adjusted serum HBsAg values demonstrated a nearly perfect AUC in both HBeAg(+) and HBeAg(−) CHB patients (Fig. 3E, F), which was superior to the use of serum HBsAg quantification only as a predictor of fibrosis severity [11]. Integrated application of serum HBsAg quantification data and ABT values may be necessary for precise prediction. It should be noted that the Ludwig scoring system was adopted in the study by Herold *et al.* [13], while the Scheuer scoring system was used in our investigation. However, the definitions of S1 to S4 are similar in these two systems (Table S1). Therefore, use of mean ABT values presented in the study by Herold *et al*. [13] was acceptable in our investigation.

We also found that there was a positive correlation between tissue HBsAg and the severity of fibrosis for all of the enrolled patients, and more particularly for HBeAg(+) patients. Using immunohistochemistry, tissue HBsAg was determined from the percentage and the strength of staining in hepatocytes. The number of viable hepatocytes has no effect on the expression of tissue HBsAg; thus, the positive correlation of tissue HBsAg with the severity of fibrosis further confirmed the relationship that we observed between ABT-adjusted serum HBsAg levels and the stage of fibrosis. Therefore, it seemed reasonable to deduce that the capacity for synthesis of HBsAg by individual hepatocyte is enhanced in cases with moderate to severe fibrosis.

Compared with recent studies [11], [12], several key differences were exhibited in our study: (1) ABT was adopted to adjust serum HBsAg levels and more precise prediction of moderate to severe fibrosis was achieved; (2) correlation between tissue HBsAg and fibrosis severity was analyzed, which further supported the relationship between ABT-adjusted serum HBsAg and the stage of fibrosis. Thus, it was helpful to elucidate the actual value of serum HBsAg; (4) 58% of the enrolled patients were HBeAg (+), which was differed markedly from the study of Martinot-Peignoux *et al*. [11]. It is well known that there is significant heterogeneity in the genomes of HBeAg(−) HBV, which may hinder the generalization of the conclusions derived from HBeAg(−) patients. Future research with long-term follow-up in humans and laboratory animal-based research using stratification according to serum HBsAg levels are indispensable for exploring the roles of HBsAg in liver fibrosis.

## Supporting Information

Figure S1 **Correlation between immunohistochemistry grade of tissue HBsAg and stage of fibrosis.** (A–C) grade of tissue HBsAg in CHB patients stratified according to the stage of fibrosis in all enrolled patients, HBeAg(+) patients, and HBeAg(−) patients, respectively. Stage of fibrosis was determined according to the Scheuer scoring system.
(TIF)

Table S1 **Definition of fibrosis stage in Ludwig scoring system and Scheuer scoring system.**
(DOCX)
