# Supplementary figures and images for: Enhanced HBsAg Synthesis Correlates with Increased Severity of Fibrosis in Chronic Hepatitis B Patients
Source: PLoS One. 2014 Jan 31;9(1):e87344. doi: 10.1371/journal.pone.0087344 (PMC3909099; doi:10.1371/journal.pone.0087344)

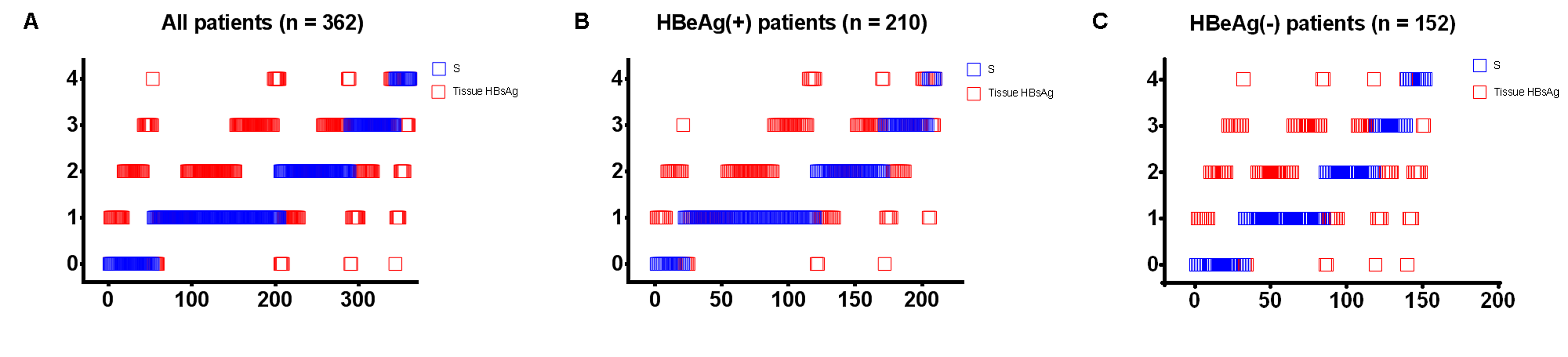

Supplement: Figure S1 — Correlation between immunohistochemistry grade of tissue HBsAg and stage of fibrosis. (A–C) grade of tissue HBsAg in CHB patients stratified according to the stage of fibrosis in all enrolled patients, HBeAg(+) patients, and HBeAg(−) patients, respectively. Stage of fibrosis was determined according to the Scheuer scoring system. (TIF) [file pone.0087344.s001.tif]
