# Supplementary material for: Enhanced HBsAg Synthesis Correlates with Increased Severity of Fibrosis in Chronic Hepatitis B Patients
Source: PLoS One. 2014 Jan 31;9(1):e87344. doi: 10.1371/journal.pone.0087344 (PMC3909099; doi:10.1371/journal.pone.0087344)
Supplement: Table S1 — Definition of fibrosis stage in Ludwig scoring system and Scheuer scoring system. (DOCX) [file pone.0087344.s002.docx]

**Supplementary Table 1.** Definition of fibrosis stage in Ludwig scoring system and Scheuer scoring system.

|  | Ludwig system | Scheuer system |
| --- | --- | --- |
| S0 | NA^*^ | None |
| S1 | No fibrosis or fibrosis confined to enlarged portal tracts | Enlarged, fibrotic portal tracts |
| S2 | Periportal fibrosis or portal-to-portal septa but intact architecture | Periportal or portal-portal septa but intact architecture |
| S3 | Septal fibrosis with architectural distortion; no obvious cirrhosis | Fibrosis with architectural distortion but no obvious cirrhosis |
| S4 | Probable or definite cirrhosis | Probable or definite cirrhosis |

^*^In Ludwig scoring system there is no stage of S0.
